# A Comparative Study of Hematological Parameters of Endurance Runners at Guna Athletics Sport Club (3100 Meters above Sea Level) and Ethiopian Youth Sport Academy (2400 Meters above Sea Level), Ethiopia

**DOI:** 10.1155/2021/8415100

**Published:** 2021-11-24

**Authors:** Zelalem Tilahun Muche, Diresibachew Haile Wondimu, Milkessa Bayissa Midekssa, Endeshaw Chekol Abebe, Teklie Mengie Ayele, Ediget Abebe Zewdie

**Affiliations:** ^1^Department of Medical Physiology, College of Health Sciences, Debre Tabor University, Debre Tabor, Ethiopia; ^2^Department of Medical Physiology, College of Health Sciences, Addis Ababa University, Addis Ababa, Ethiopia; ^3^Department of Sport Science, Sport Science Academy, Haramaya University, Haramaya, Ethiopia; ^4^Department of Medical Biochemistry, College of Health Sciences, Debre Tabor University, Debre Tabor, Ethiopia; ^5^Department of Pharmacy, College of Health Sciences, Debre Tabor University, Debre Tabor, Ethiopia

## Abstract

**Introduction:**

Endurance running performance is dependent upon hematological, physiological, anthropometrical, diet, genetic, and training characteristics. Increased oxygen transport and efficiency of tissue in extracting oxygen are the major determinants to competitions that require endurance. Thus, altitude training is often employed to increase blood oxygen-carrying capacity to improve sea-level endurance performance. This study aimed to compare hematological parameters of endurance runners' training at different clubs with different altitudes (Guna Athletics Sport Club at Guna (3100 meter above sea level) and Ethiopian Youth Sport Academy at Addis Ababa (2400 meter above sea level)).

**Methods:**

A comparative cross-sectional study was conducted at GASC and EYSA. Data were collected from a total of 102 eligible study subjects (26 runners and 25 controls at Guna and 26 runners and 25 controls at Addis Ababa) from May to October 2019. About 3 ml of the venous blood was drawn from the antecubital vein by aseptic procedure and analyzed using a hematology analyzer (DIRUI BCC-3000B, China). One-way ANOVA and independent-sample *t*-tests were used to compare means.

**Result:**

Male runners in Guna had significantly higher hemoglobin (Hgb), mean corpuscular hemoglobin (MCH), mean corpuscular hemoglobin concentration (MCHC), and white blood cell (WBC) count than male runners in Addis Ababa. Besides, female runners in Guna had significantly higher MCH and MCHC than female runners in Addis Ababa. However, there were no significant differences between Guna and Addis Ababa runners in red blood cell (RBC) count, Hct, MCV, and platelet count in both sexes, while Hgb and WBC count in females.

**Conclusion:**

Decisively, Guna Athletics Sport Club endurance runners had significantly higher hematological parameters than Ethiopian Youth Sport Academy endurance runners. This provides invaluable information for coaches and sport physicians to monitor the hematological profile and the health status of an athlete living and training at different altitudes.

## 1. Introduction

Running is one of the most popular sporting events worldwide, and running events range from sprints of 60 meters (m) to ultramarathons covering greater than 42.195 kilometers [1]. The International Association of Athletics Federation classified running as short distance (below 800 m), middle distance (800–3000 m), and long distance (3000-ultramarathon (>marathon)). Endurance running is highly dependent on the aerobic capacity and running economy [1].

Altitude can be classified as near sea level (<500 m), low altitude (500–2,000 m), moderate altitude (2,000–3,000 m), high altitude (3,000–5,500 m), and extreme altitude (>5,500 m) above sea level [2]. As altitude increases, atmospheric pressure decreases, and the partial pressure of oxygen also decreases, thus the amount of oxygen available for delivery to exercising tissues will reduce [3, 4].

Altitude training is aimed at increasing the oxygen-carrying capacity of blood to improve sea-level endurance performance in athletes. Increasing erythropoietin production in hypoxia, a hormone-stimulating erythropoiesis, is a key factor in the achievement of enhanced oxygen-carrying capacity of the blood. Rates of erythropoietin production and erythropoiesis depend on the duration and degree of exposure to hypoxia. Furthermore, many other factors may affect the hematological response to altitude training [5]. Currently, altitude training has become part of the standard training protocol in many aerobic sports to increase endurance performance in athletes or to acclimatize before competitions at altitude or before ascending to altitude [3, 6, 7]. Acute or chronic exposure of the human body to a hypoxic environment induces several adaptations that can lead to improved athletes' performance at sea level. The mechanisms to improve exercise performance including hematological [8–10], cardiovascular, or ventilatory changes were induced by altitude training. However, altitude training can also lead to improved muscle buffering capacity, enhanced capillary density, and muscle mitochondrial volume [11–14].

Previous studies have shown that after altitude training of LH-TH or LH-TL there were increased red cell mass, total hemoglobin mass, reticulocytes, red blood cell (RBC) count, hemoglobin (Hgb), and hematocrit (Hct) from pre-altitude value [11, 15–20]. However, other studies did not show an increase in red cell mass, total hemoglobin mass, reticulocytes, RBC count, Hgb, and Hct after altitude training [21, 22]. Individual variation in response to altitude exposure is an important factor that needs to be accounted for when planning altitude training [5, 10, 15, 23].

There are different models of altitude training: live high-train high (LH-TH), live high-train low, live low-train high, and live high-train low and high [4, 6]. Among them, the LH-TH method is the traditional concept of altitude training, practiced by athletes in East Africa. In this model, athletes live and train at moderate altitudes 2,000–3,000 m above sea level that is thought to stimulate hematological and nonhematological responses [6, 7, 24]. This method is still in use today in particular in countries with natural altitude environments, including Kenya and Ethiopia [7, 17]. Athletes employing LH-TH are not able to train at an equivalent or near-equivalent intensity as at sea level [7, 16, 17, 25]. Altitudes that are too low are associated with the inadequate erythropoietic response [7, 16, 25]. Generally, in living high-training high approach, the optimal altitude to improve exercise performance is between 2,000 and 2,500 m above sea level [4, 26].

Different hematological, physiological, anthropometrical, diet, genetic, motivation, and training characteristics influence endurance running performances, depending on the length and duration of the performance training [27–34]. Factors that have been proposed to explain the dominance of East African athletes, particularly the success of the Kenyan and Ethiopian distance runners, include genetic predisposition, favorable skeletal-muscle-fiber composition, oxidative enzyme profile, development of high maximal oxygen uptake, relatively high Hct and Hgb, good metabolic “economy”, traditional Kenyan/Ethiopian diet, living and training at altitude, and motivation to achieve economic success [29, 30, 33, 35–39].

There is increasing support for the role of hematological variables like RBC count, Hgb, Hct, total hemoglobin mass, and blood volume in determining endurance performance [29, 31, 40, 41]. Since the availability of oxygen in skeletal muscle impacts endurance performance, it is essential to monitor hematological parameters to detect the oxygen transport capacity of endurance athletes [42, 43].

Ethiopia has many altitudinous areas ranging from 1500 m to 4550 m above sea level; however, athletes are emerging from a specific area and population particularly from Arsi and Shewa [29, 30, 39]. There are no published data to support or refute that Hgb and total blood volume in Ethiopian athletes are uniquely different from other elite running populations [29]. Unique hematological fluctuations observed in an athlete population provide invaluable information to the sports physician monitoring the health status of an athlete [10].

To the best of our knowledge, no study compared hematological parameters of Ethiopian endurance runners training at various clubs that are located at different altitudes. Therefore, this study compared hematological parameters in endurance runners of Guna Athletics Sport Club, which is located in Northern Ethiopia at 3100 m above sea level, and Ethiopian Youth Sport Academy, which is located in the central part of Ethiopia (Addis Ababa, the capital city of Ethiopia) at 2400 m above sea level. The two clubs use a live high-train high model, yet the altitude varies (3100 m vs 2400 m above sea level).

Our hypothesis was there is no significant difference in hematological parameters between Guna Athletics Sport Club and Ethiopian Youth Sport Academy endurance runners.

## 2. Methods

### 2.1. Study Area, Period, and Design

A comparative institutional-based cross-sectional study design was employed to conduct the study from May to October 2019 in two training camps in Ethiopia, Guna Athletics Sport Club and Ethiopian Youth Sport Academy. Guna Athletics Sport Club is located in the Amhara region, South Gondar zone, near Guna Mountain (nearly 4200 m above sea level), which is 695 km far from Addis Ababa. This training camp is particularly situated at an altitude of 3045 m above sea level, and routine training takes place at 3100 m above sea level. The second study area was Ethiopian Youth Sport Academy, which is located in Addis Ababa, at approximately an altitude of 2400 m above sea level.

### 2.2. Study Population

All endurance runners in GASC and EYSA fulfilling the eligibility criteria were taken as the study population.

### 2.3. Sampling Procedures

A total of 102 study subjects participated in this study. A total of 26 endurance runners from each training camp and 25 matched nonathletes were recruited outside each training camp. Based on sex, 18 male and 8 female endurance runners were involved from each camp, and 18 male and 7 matched female nonathletes were involved from each camp. A convenient nonprobability sampling technique was used to select eligible study subjects.

### 2.4. Eligibility Criteria

Both male and female athletes who were middle- and long-distance runners ranging from 800 m to marathon, as well as those in the age range of 15 to 35 years were included in the study. However, athletes in Addis Ababa whose root is from northern training camps (Amhara region); athletes in Guna whose root is from Addis Ababa (Oromia region); athletes who were reported to have known cancer, kidney disease, liver disease, HIV/AIDS, cardiac diseases, anemia, and respiratory diseases (like asthma); smokers; athletes trained less than 5 days; athletes on vacation; and pregnant during the data collection period were excluded from the study.

### 2.5. Study Variables

In the present study, running performance or the International Association of Athletics Federation score (IAAF score) was taken as the dependent variable, while sociodemographic variables such as age, sex, marital status, and religion, anthropometric parameters, including weight, height, and body mass index, and hematological parameters such as RBC count, Hct, Hgb, MCV, MCH, MCHC, WBC count, and platelet count were considered independent variables.

### 2.6. Operational Definitions

**Elite athlete:** professional runner who is competing at the national or international level. **Endurance runners**: runners who run from 800 m to ultramarathon. **Middle-distance running**: running covering the distance from 800 m to 3000 m. **Long-distance running**: running covering the distance from 3000 m to ultramarathon. **International Association of Athletics Federation score (IAAF score):** it is the measure of an athlete's performance, and this score can be used to determine the result score of performance for the world rankings, to evaluate competitions, and to establish the best athlete award in a specific competition [44]. **Total hemoglobin mass:** it is the absolute mass of circulating hemoglobin in the body.

### 2.7. Data Collection Procedures

After informed consent, sociodemographic data were collected by using structured questionnaires from the selected participants through face-to-face interviews. Then, the height of the study participants was measured without shoes using a stadiometer and rounded to the nearest one cm, whereas the weight of subjects was measured using a weighing scale to the nearest 0.1 kg with light clothing, without phones and shoes or any encumbrance that could alter their appropriate weight. Body mass index (BMI) was calculated by dividing weight (in kg) by height (in meters) squared. By following the aseptic procedure, about 3 ml of the venous blood sample was drawn from the antecubital vein of each participant by a trained and qualified laboratory technologist after overnight fasting. The blood sample in the Guna training camp was collected using EDTA-coated vacutainer tubes, and it was then transported in sealed boxes to Bahir Dar within an hour of blood collection and at room temperature. The laboratory analysis was done at Afilas Primary Hospital in Bahir Dar using (DIRUI BCC-3000B; China) a hematology analyzer within 5 hours of the blood sample collection. Similarly, samples at the Addis Ababa training camp were collected using EDTA-coated vacutainer tubes, and then the laboratory analysis was done within an hour of sample collection at the clinic in the center using a similar automated blood analyzer.

Performance of runners was measured using the IAAF score, which was taken from the IAAF score table (2017) by using personal best time. It was also checked by the online IAAF scoring calculator. Tables are normally valid for performances worth between 0 and 1400 points [44].

### 2.8. Data Processing and Statistical Analysis

The data collected were coded, cleaned, entered, and analyzed using Statistical Package for Social Sciences (SPSS), version 25.0. Categorical variables were presented using frequency and percent, whereas continuous variables were summarized using mean (*x̅*) and standard deviation (SD). The analysis of the differences in means of study variables was evaluated using an independent-sample *t*-test and one-way ANOVA. We used Levene's test to assess the homogeneity of variance, and the Tukey and Games–Howell post hoc tests were used if Levene's test was nonsignificant and significant, respectively. Those variables with a *p*-value of <0.05, at a 95% confidence interval (CI), were considered statistically significant. The result of males and females were summarized separately.

## 3. Results

### 3.1. Sociodemographic Data

The total study participants were 102 (51 from Guna Athletics Sport Club (Guna) and 51 from Ethiopian Youth Sport Academy (Addis Ababa), among them 72 (70.6%) were males and 30 (29.4%) were females. There were 52 athletes (26 from each camp) and 50 nonathletes (25 from each camp). Out of 26 athletes in Guna (AG), 18 (69.2%) were males and 8 (30.8%) were females. Also from 25 nonathletes in Guna (NAG), 18 (72%) and 7 (28%) were males and females, respectively. Athletes in Addis Ababa (AAA) were 26, of these 18 (69.2%) were males and 8 (30.8%) were females. Nonathletes in Addis Ababa (NAAA) were 25, among them 18 (72%) and 7 (28%) were males and females, respectively.

Among 102 participants, 51 (50%) were from Oromia, and 51 (50%) were from the Amhara region. The mean ages of study groups for both sexes are presented in Table 1. The majority of male and female subjects in both groups belonged to the age bracket of 15–19 and 20–24 years, respectively. One-way ANOVA showed there were no significant differences in mean ages between AG vs AAA, AG vs NAG, and AAA vs NAAA for both sexes (Table 1). Moreover, there were no significant differences in height, weight, and BMI between AG and AAA in both sexes. Regarding marital status, the majority of AG 25 (96.2%), AAA 23 (88.5%), NAG 23 (92%), and NAAA (84%) were single. The majority of AG 20 (76.9%), AAA 12 (46.2%), NAG 13 (52%), and NAAA 12 (48%) attended secondary and preparatory school. The majority of AG (96.2%), NAG (100%), and NAAA (64%) were orthodox Christians; however, most of the athletes in Addis Ababa (46.2%) were protestant Christians.

Nearly all study participants of AG (84.6%), AAA (88.5%), and NAG (100%) go to school by walking or running. Nevertheless, almost half of NAAA (48%) go to school by using public transportation. Most athletes in both study groups stated that they chose running to boost their economy/income (AG vs AAA: 92.3%, 96.2%), and a very few aspired to be famous (AG vs AAA: 7.7%, 3.8%). Among 52 athletes, 20 (38.5%) were middle-distance runners and 32 (61.5%) were long-distance runners.

### 3.2. Performance of Athletes

The mean performance/IAAF scores of male athletes of Guna and Addis Ababa were 947 ± 85.6 and 940 ± 85.4 points, respectively. Female athletes in Guna and Addis Ababa had IAAF scores of 1011.3 ± 85 and 973.9 ± 118, respectively. There were no significant differences between the performance of AG and AAA in both sexes.

### 3.3. Hematological Parameters

**Figure 1:**
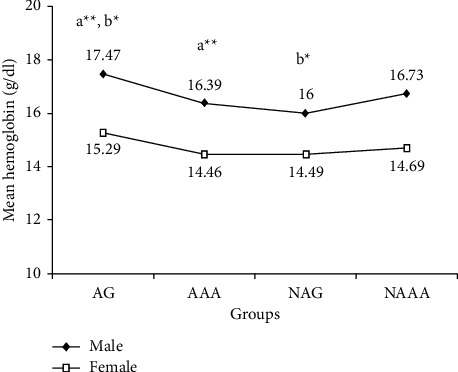
Line graph showing hemoglobin concentration of study groups for both groups, Guna, Addis Ababa, Ethiopia, 2020. AG, athletes in Guna; NAG, nonathletes in Guna; AAA, athletes in Addis Ababa; NAAA, nonathletes in Addis Ababa. (a) AG vs AAA; (b) AG vs NAG; ^*∗*^*p* < 0.05; ^*∗∗*^*p* < 0.001.

**Figure 2:**
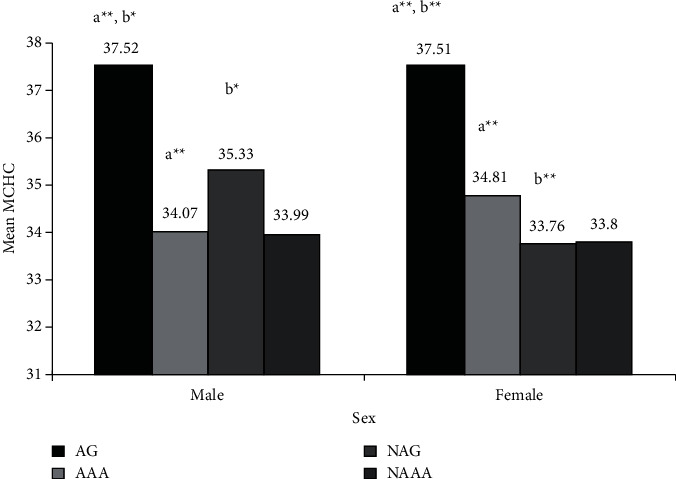
Bar graphs showing mean corpuscular hemoglobin concentration (g/dl) of study groups for both sexes, Guna, Addis Ababa, Ethiopia, 2020. (a) AG vs AAA; (b) AG vs NAG; ^*∗*^*p* < 0.01; ^*∗∗*^*p* < 0.001.

Comparison of RBC count, Hct, mean corpuscular volume (MCV), mean corpuscular hemoglobin (MCH), white blood cell (WBC) count, and platelet count between study groups are presented in Table 2; Hgb and mean corpuscular hemoglobin concentration (MCHC) are presented in Figures 1 and 2, respectively. There were no significant differences in mean RBC count between male AG vs AAA, AAA vs NAAA, and between all-female study groups. However, male AG had a significantly higher RBC count than NAG (AG vs NAG: 5.58 ± 0.3 vs 5.1 ± 0.4 × 1012/L; *p* < 0.01; (Table 2)). Male AG had significantly higher Hgb than AAA and NAG (AG vs AAA: 17.47 ± 0.9 vs 16.39 ± 0.9 g/dl; *p* < 0.01; AG vs NAG: 17.47 ± 0.9 vs 16 ± 1.6 g/dl; *p* < 0.05). Nevertheless, there were no significant differences in Hgb between male AAA vs NAAA and between female study groups (Figure 1).

We found that there were no significant differences in Hct between AG vs AAA, AAA vs NAAA in both sexes, while AG vs NAG in females. However, male AG had significantly higher Hct than NAG (AG vs NAG: 49.03 ± 2 vs 45.32 ± 3.1 g/dl; *p* < 0.01). MCV was not significantly different between study groups in both sexes. AG had significantly higher MCH than AAA for both sexes (AG vs AAA, male: 33.2 ± 1.2 vs 29.5 ± 0.9 pg; *p* < 0.001; and female: 32.8 ± 1.6 vs 29.7 ± 1.6 pg; *p* < 0.01). Similarly, AG had significantly higher MCH than NAG in both sexes (AG vs NAG, male: 33.2 ± 1.2 vs 31.4 ± 2.2 pg; *p* < 0.05; and female: 32.8 ± 1.6 vs 29.9 ± 2.1 pg; *p* < 0.05). However, there were no significant differences in MCH between AAA vs NAAA in both sexes (Table 2).

As shown in Figure 2, AG had significantly higher MCHC than AAA in both sexes (AG vs AAA, male: 37.52 ± 0.9 vs 34.07 ± 0.8 g/dl; *p* < 0.001; and female: 37.51 ± 0.6 vs 34.81 ± 1.5 g/dl; *p* < 0.001). Furthermore, AG had significantly higher MCHC than NAG (AG vs NAG, male: 37.52 ± 0.9 vs 35.33 ± 2.2 g/dl, *p* < 0.01; and female: 37.51 ± 0.6 vs 33.76 ± 0.9; *p* < 0.001). However, there were no significant differences in MCHC between AAA vs NAAA in both sexes (Figure 2). Male AG had significantly higher WBC count than male AAA (AG vs AAA: 6.13 ± 1.6 vs 4.78 ± 1.3 × 10^9^/L; *p* < 0.05). However, there were no significant differences in WBC count between male AG vs NAG, AAA vs NAAA, and between all-female study groups. There were no significant differences in platelet count between male study groups and female AG vs AAA, AAA vs NAAA. However, female AG had a significantly higher platelet count than NAG (AG vs NAG: 297.88 ± 70.15 vs 209.71 ± 52.19 × 10^9^/L; *p* < 0.05 (Table 2)).

## 4. Discussion

The present study is designed to compare the hematological parameters of endurance runners training at Guna Athletics Sport Club who were living and training at 3100 m above sea level and Ethiopian Youth Sport Academy who were living and training at ∼2400 m above sea level. Our study indicated that male AG had significantly higher Hgb, MCH, MCHC, and WBC count than male AAA. Besides, female AG had significantly higher MCH and MCHC than female AAA.

This study showed there was no significant difference in mean RBC count between male AG and male AAA. This is similar to a study that showed male Eritrean and Spaniard runners had similar RBC counts [45]. The male mean RBC count of both AG and AAA were almost similar to the RBC count of male professional Ethiopian runners, Southern Ethiopian soccer players, and cyclists [11, 46, 47]. Besides, there was no significant difference in RBC count between female AG and AAA. This is in contrast to a study that revealed female runners had a significantly lower RBC count than cyclists [48]. This difference might be due to variation in the discipline of sports they specialized for (running and cycling). It can also be due to variation in plasma volume expansion, which may be higher in running than cycling. Furthermore, both female AG and AAA runners had the RBC count similar to female Ethiopian professional runners [47].

In the present study, Hgb was significantly higher in male AG than AAA. This is in line with a study that showed that Kenyan runners have significantly higher Hgb than Scottish runners before erythropoietin administration [49]. However, it is different from other studies, which found no significant differences in Hgb between Eritrean and Spaniard runners [45], between Kenyan and Scandinavian runners [50], and between Kenyan and German endurance runners [41]. The reason for higher Hgb in AG than AAA may be due to AG were more sweaters than others since living and training at a high altitude (3100 m) may induce perspiration and urination more than AAA.

The mean Hgb of male athlete runners was higher than other male professional Ethiopian runners, elite male Kenyan distance runners, Eritrean endurance runners, and Southern Ethiopian soccer players [41, 45, 47, 49, 51–53]. The observed difference can be ascribed to differences in the rate of sweating, altitude (3100 m above SL), level of athletes, and plasma volume expansion. The observed higher Hgb of male Guna runners may increase the blood viscosity, which will increase the cardiac burden and affects performance. There was no significant difference in Hgb between female Guna and Addis Ababa runners. Mean Hgb concentration of both female AG and AAA were almost similar with female Ethiopian professional runners (15 ± 0.9 g/dl) [47] but higher than Kenyan runners (13.4 ± 0.4 g/dl) [52]. This difference may be due to variations in the level of athletes, rate of sweating, and altitude.

A study done by Beall et al. on native altitude residents (3,800–4,065 m above SL) showed that Tibetan males had significantly lower Hgb than Aymara males (15.6 vs 19.2 g/dl). Furthermore, Tibetan females had lower Hgb than Aymara females (14.2 vs 17.8 g/dl) [54]. However, Beall and her colleagues who studied on high-altitude natives of Ethiopia (3500 m) near the Simien Mountains in the Amhara region found an average Hgb of 15.9 and 15.0 g/dl for males and females, respectively. The mean Hgb of Ethiopian high-altitude natives was similar to U.S. blacks who were sea-level natives [55]. Thus, Ethiopian highlanders maintain venous Hgb within the ranges of sea-level populations, despite decreases in the partial pressure of oxygen at high altitudes. Conversely, Scheinfeldt et al. (2012) compared the Hgb of 28 male Amhara (living at 3,202 m in Debele, near Debre Birhan, Ethiopia), 8 Aari men in Ethiopia (living at 1,407 m), and 7 Hamer men in Ethiopia (living at 1,097 m), which showed there was a significant increase in Hgb in the Amhara (16.4 g/dl) relative to the Aari (14.8 g/dl) and Hamer (12.4 g/dl) population [56].

Our study also showed no significant difference between Hct of male AG and AAA. These values are similar to Kenyan distance runners [41, 51] but higher than Southern Ethiopian soccer players, Eritrean runners, cyclists, Kenyan runners, and swimmers at 2300 m [11, 19, 45, 46, 49, 52, 53]. This might be due to differences in altitude, level of athletes, and rate of sweating. In this study, there is no significant difference in mean Hct between female Guna and Addis Ababa runners (42.9 ± 2.1 and 41.66 ± 1.9%). This is similar to female Kenyan runners who have Hct of 41.2 ± 1.2% [52].

This study showed AG had significantly higher MCH and MCHC than AAA in both sexes. This may be due to AG having higher Hgb than AAA, hence, higher MCH and MCHC.

Male AG had significantly higher Hgb and Hct than NAG; this is similar to other studies [57–59], but different from studies that showed similar Hgb and Hct between athletes and nonathletes [60, 61]. This overall variation in Hgb and Hct might be due to differences in the level of athletes, dose and duration of hypoxia exposure, plasma volume level, training frequency and intensity, age of study subjects, and nutrition. Physical training and duration of exercise have major importance in the adaptation of the blood cell system [58, 59, 62].

### 4.1. Strength and Limitation of the Study

It was the first study that compared hematological parameters of endurance runners training at two training camps located at different altitudes and different origins but within the same country. We have involved control groups for both athletes at GASC (Guna) and EYSA (Addis Ababa). Despite the strengths, we did not measure VO2 max, fractional utilization of VO2 max, running economy, nutrition genetics, and total hemoglobin mass.

## 5. Conclusion

Decisively, there were significant differences in hematological parameters between AG and AAA in both sexes. Guna Athletics Sport Club endurance runners had significantly higher hematological parameters than Ethiopian Youth Sport Academy endurance runners. This study provides invaluable information for coaches and sport physicians to monitor the hematological profile and the health status of an athlete living and training at different altitudes.

## Figures and Tables

**Table 1:** Comparison of age, height, weight, and BMI of study groups for both sexes using one-way ANOVA, Guna, Addis Ababa, Ethiopia, 2020.

Variables	AG	AAA	NAG	NAAA
*Male*				
Age (yrs)	23.3 ± 3.7	24.2 ± 3.9	21.2 ± 3.7	26.3 ± 3.3
Height (cm)	169.9 ± 4.1 **b**^*∗∗*^	172 ± 8.9	162.6 ± 6.4 **b**^*∗∗*^	169.8 ± 6.5
Weight (kg)	56.3 ± 6.3	58.8 ± 6.1	53.5 ± 7	64.6 ± 7.9
BMI (kg/m^2^)	19.4 ± 1.6	19.9 ± 1.6 **c**^*∗*^	20.2 ± 1.6	22.4 ± 2.7 **c**^*∗*^

*Female*				
Age	19.9 ± 1.5	20.4 ± 3.4	21.9 ± 3.4	23.4 ± 2.1
Height	159.9 ± 5	164 ± 5	159.3 ± 10	165.6 ± 5.8
Weight	46.8 ± 3	51.4 ± 6.7	52.3 ± 4.6	60 ± 9.7
BMI	18.3 ± 1.3	19.2 ± 1.7	20.7 ± 1.6	21.9 ± 3.4

Abbreviations: AG, athletes in Guna; NAG, nonathletes in Guna; AAA, athletes in Addis Ababa; NAAA, nonathletes in Addis Ababa; **b**, AG vs NAG; **c**, AAA vs NAAA; ^*∗*^*p* < 0.05; ^*∗∗*^*p* < 0.01.

**Table 2:** One-way ANOVA result for the comparison of RBC count, Hct, MCV, MCH, WBC count, and platelet count between study groups for both sexes, Guna, Addis Ababa, Ethiopia, 2020.

Variables	AG	AAA	NAG	NAAA
*Male*				
RBC × 1012/L	5.58 ± 0.3 b^*∗∗*^	5.56 ± 0.3	5.11 ± 0.4 b^*∗∗*^	5.72 ± 0.5
Hct (%)	49.03 ± 2 b^*∗∗*^	48.11 ± 2.7	45.32 ± 3.1 b^*∗∗*^	49.29 ± 3.4
MCV (fl)	88.5 ± 2.6	86.6 ± 2.5	89.2 ± 2.6	86 ± 3
MCH (pg)	33.2 ± 1.2 a^*∗∗∗*^ b^*∗*^	29.5 ± 0.9 a^*∗∗∗*^	31.4 ± 2.2 b^*∗*^	29.2 ± 1
WBC × 109/L	6.13 ± 1.6a^*∗*^	4.78 ± 1.3a^*∗*^	7.98 ± 2.6	5.8 ± 1.4
Platelet × 109/L	266.3 ± 76.7	241 ± 38.5	242.1 ± 68.5	233.6 ± 60.9

*Female*				
RBC × 1012/L	4.92 ± 0.2	4.87 ± 0.3	4.81 ± 0.1	5.05 ± 0.3
Hct (%)	42.88 ± 2.1	41.66 ± 1.9	42.91 ± 3	43.44 ± 3.2
MCV (fl)	87.5 ± 41	85.5 ± 2.9	89.2 ± 5	86.1 ± 3.5
MCH (pg)	32.8 ± 1.6 a^*∗∗*^ b^*∗*^	29.7 ± 1.6 a^*∗∗*^	29.9 ± 2.1 b^*∗*^	29.1 ± 1.1
WBC × 109/L	6.14 ± 2	5.03 ± 1.2	7.74 ± 1.6	6.76 ± 2
Platelet × 109/L	297.88 ± 70.15 b^*∗*^	256.13 ± 42.96	209.71 ± 52.19 b^*∗*^	291 ± 55.46

AG, athletes in Guna; NAG, nonathletes in Guna; AAA, athletes in Addis Ababa; NAAA, nonathletes in Addis Ababa; **a**, AG vs AAA; **b**, AG vs NAG; **c**, AAA vs NAAA; ^*∗*^*p* < 0.05; ^*∗∗*^*p* < 0.001; ^*∗∗∗*^*p* < 0.001.

## Data Availability

All the data sets generated and analyzed during the study are included in the text.

## Abbreviations

AAA: Athletes in Addis Ababa
AG: Athletes in Guna
EDTA: Ethylenediaminetetra acetic acid
EYSA: Ethiopian Youth Sport Academy
GASC: Guna Athletics Sport Club
Hct: Hematocrit
Hgb: Hemoglobin
IAAF: International Association of Athletics Federation
LH-TH: Live high-train high
MCH: Mean corpuscular hemoglobin
MCHC: Mean corpuscular hemoglobin concentration
MCV: Mean corpuscular volume
NAAA: Nonathletes in Addis Ababa
NAG: Nonathletes in Guna
RBC: Red blood cell
WBC: White blood cell.
